# Characterization and phylogenetic analysis of the complete mitochondrial genome of sun loach (*Yasuhikotakia eos*)

**DOI:** 10.1080/23802359.2020.1842263

**Published:** 2021-01-05

**Authors:** Xiaoge Yang, Yuxi Lian, Minmin Chen, Xuequn Li, Daoping Yu

**Affiliations:** a Research Center of Aquatic Organism Conservation and Water Ecosystem Restoration in Anhui Province, College of Life Science, Anqing Normal University, Anqing, PR China; b Cambodian First Unite Construction Group, Phnom Penh, Cambodia

**Keywords:** *Yasuhikotakia eos*, mitochondrial genome, Botiinae, phylogeny

## Abstract

The complete mitochondrial genome of the sun loach (*Yasuhikotakia eos*) was determined based on Illumina data in this study. The result showed that the closed double-stranded circular mitogenome was 16,738 bp in total length (GenBank accession number: MT800510) with 58.41% AT. The mitochondrial DNA consisted of 13 protein-coding genes (PGCs), 22 transfer RNA (tRNA) genes, 2 ribosomal (rRNA) genes, and 1 non-coding control region. Phylogenetic analysis suggested that *Y*. *eos* was most closely related to its congener *Y*. *modesta*. This work provides molecular information for further research on species identification and evolutionary relationships.

The sun loach (*Yasuhikotakia eos*), belonging to the subfamily Botiinae (Cypriniformes: Cobitidae), was known to be mainly distributed in the Mekong River, Chao Phraya, and Maeklong basins in Asia (Rainboth 1996). This species is extremely aggressive as other belligerent loach species and inhabits in rapidly flowing rivers. Due to the similar morphology, *Y*. *eos* was easily confused with its sister species during the taxonomic identification, particularly for the smaller or incomplete specimen (Nalbant 2002). Here, we described the characterization of the mitochondrial genome of *Y*. *eos* and explored the phylogenetic relationship among Botiinae species, which were expected to contribute to the further studies on taxonomic resolution and phylogenetic relationships of these taxa.

*Yasuhikotakia eos* was collected in Mekong River (12°46′33.2′′N, 105°57′29.6′′E), Cambodia in December 2019, and were deposited in Mekong Fish Herbarium of Cambodian First Unite Construction Group (Specimen ID: MK2019040). Tiny tail fin tissues were obtained and preserved in 95% ethanol. Total genomic DNA was extracted with a QIAamp DNA mini kit (Qiagen, Germany) following the manufacturer’ instructions. After monitored, the genomic DNA was sequenced through Illumina Hiseq 2500 platform (Illumina, San Diego, CA), and the mitogenome was assembled by metaSPAdes software (Nurk et al. 2017) using *Yasuhikotakia morleti* as reference.

The complete mitochondrial genome of *Y*. *eos* was a circular molecule with 16,738 bp in total length (GenBank accession number: MT800510). The overall nucleotides composition was 32.34%, 25.98%, 26.31%, and 15.28% for A, T, C, and G respectively, which showed a bias toward A and T (58.41%). Similar to the typical mitogenome of vertebrates, *Y*. *eos* also contained a set of 13 protein-coding genes (PCGs), 22 transfer RNA (tRNA) genes, 2 ribosomal RNA (rRNA) genes, and 1 non-coding control region (Supplementary Figure S1) (Ma et al. 2014; Wang et al. 2019). Among these 37 genes, 9 genes (*ND6*, *tRNA^Cys^*, *tRNA^Tyr^*, *tRNA^Ser^*, *tRNA^Ala^*, *tRNA^Gln^*, *tRNA^Glu^*, *tRNA^Asn^*, and *tRNA^Pro^*) were encoded on the light (−) strand, while the remaining 28 genes were located at the heavy (+) strand. Most of the PCGs used ATG as the initiation codon except *ND5* (started with ATA), *COI* (started with GTG), and *ND3* (started with ATT). Besides, 10 PCGs terminated with TAA codon, but *ND2*, *ND3*, and *ND4* used TAG as the stop codon. The length of 22 tRNAs ranged from 66 bp (*tRNA^Cys^*) to 76 bp (*tRNA^Lys^*). Two rRNA genes, 12S RNA (950 bp) and 16S RNA (1673 bp) were distributed between *tRNA^Phe^* and *tRNA^Leu^*, separated by *tRNA^Val^*. The D-loop region was located between *tRNA^Phe^* and *Trna^Pro^* with 1080 bp in length.

The mitochondrial genes have been widely used for species identification and inferring phylogenetic relationships so far (Frezal and Leblois 2008; Kochzius et al. 2010). Based on the 13 PCGs of *Y*. *eos* and other 18 species of Botiinae (Mao et al. 2014), a phylogenetic tree was constructed using neighbor-joining (NJ) method on Mega version 7.0 (US) with 1000 bootstrap replicates. The result showed that Botiinae was divided into two clusters (Figure 1). Furthermore, the Yasuhikotakia and the Syncrossus clustered within the same clade. *Y*. *eos* has a closest relationship with *Y. modesta*, a species of the same genus.

**Figure 1.**
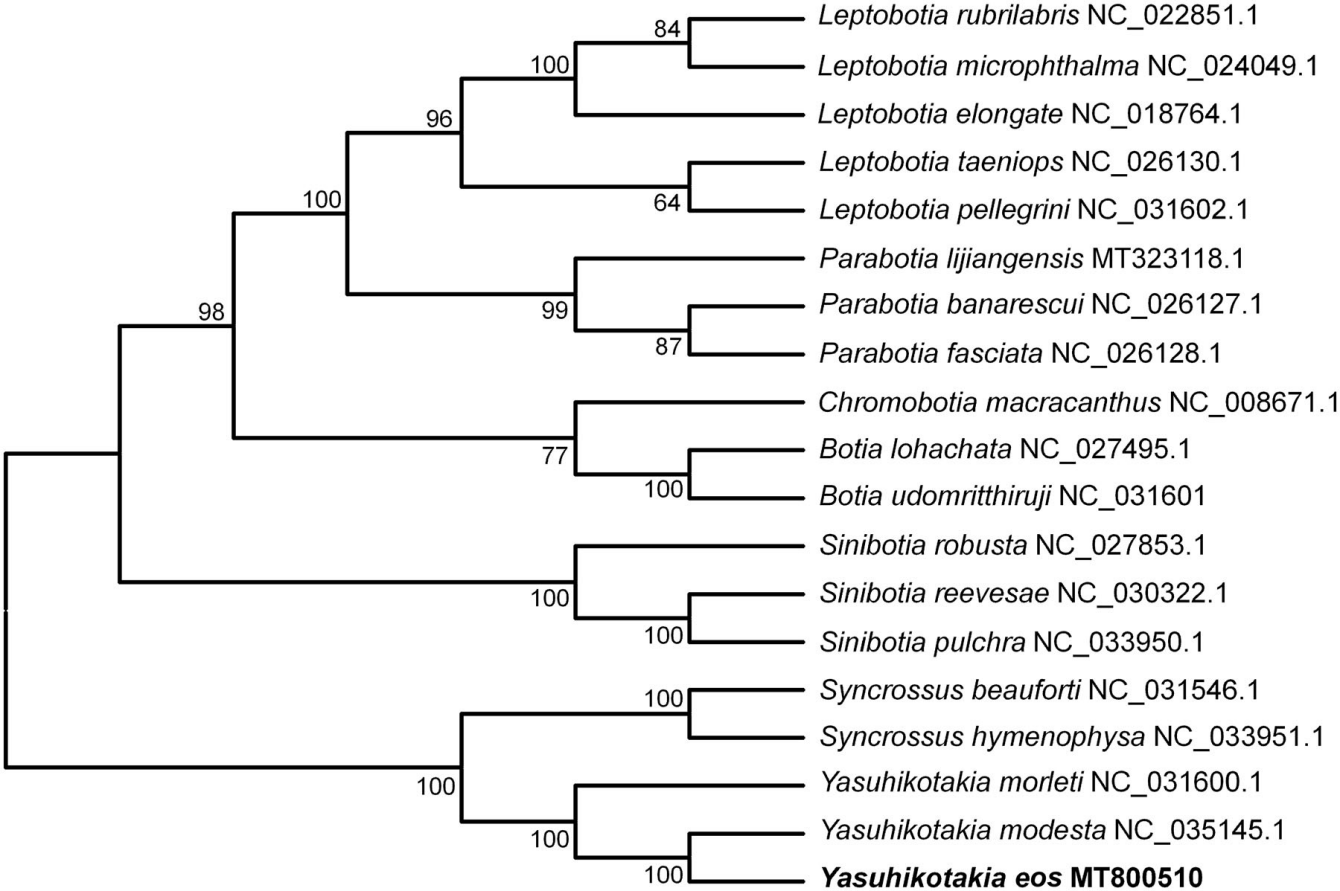
Phylogenetic analysis of 19 Botiinae species based on 13 mitochondrial PCGs by using Mega 7.0 with the neighbor-joining method (1000 replicates). Species names are followed by GenBank accession numbers.

## Data Availability

The data that support the findings of this study are openly available in the National Center for Biotechnology Information (NCBI) at https://www.ncbi.nlm.nih.gov, accession number MT800510.
